# TRAIL-R1 and TRAIL-R2 Mediate TRAIL-Dependent Apoptosis in Activated Primary Human B Lymphocytes

**DOI:** 10.3389/fimmu.2019.00951

**Published:** 2019-04-30

**Authors:** Julian Staniek, Raquel Lorenzetti, Bianca Heller, Iga Janowska, Pascal Schneider, Susanne Unger, Klaus Warnatz, Maximilian Seidl, Nils Venhoff, Jens Thiel, Cristian Roberto Smulski, Marta Rizzi

**Affiliations:** ^1^Clinic for Rheumatology and Clinical Immunology, Faculty of Medicine, Medical Center–University of Freiburg, University of Freiburg, Freiburg, Germany; ^2^Faculty of Biology, University of Freiburg, Freiburg, Germany; ^3^Department of Biochemistry, Faculty of Biology and Medicine, University of Lausanne, Lausanne, Switzerland; ^4^Center for Chronic Immunodeficiency, Faculty of Medicine, Medical Center–University of Freiburg, University of Freiburg, Freiburg, Germany; ^5^Department of Pathology, Faculty of Medicine, Medical Center–University of Freiburg, University of Freiburg, Freiburg, Germany; ^6^Medical Physics Department, Centro Atómico Bariloche, Comisión Nacional de Energía Atómica (CNEA), Consejo Nacional de Investigaciones Científicas y Técnicas (CONICET), San Carlos de Bariloche, Argentina

**Keywords:** B lymphocytes, TRAIL-R, apoptosis, human, TRAIL

## Abstract

The maintenance of B cell homeostasis requires a tight control of B cell generation, survival, activation, and maturation. In lymphocytes upon activation, increased sensitivity to apoptotic signals helps controlling differentiation and proliferation. The death receptor Fas is important in this context because genetic *Fas* mutations in humans lead to an autoimmune lymphoproliferative syndrome that is similar to lymphoproliferation observed in Fas-deficient mice. In contrast, the physiological role of TNF-related apoptosis-inducing ligand receptors (TRAIL-Rs) in humans has been poorly studied so far. Indeed, most studies have focused on tumor cell lines and on mouse models whose results are difficult to transpose to primary human B cells. In the present work, the expression of apoptosis-inducing TRAIL-R1 and TRAIL-R2 and of the decoy receptors TRAIL-R3 and TRAIL-R4 was systematically studied in all developmental stages of peripheral B cells isolated from the blood and secondary lymphoid organs. Expression of TRAIL-Rs is modulated along development, with highest levels observed in germinal center B cells. In addition, T-dependent and T-independent signals elicited induction of TRAIL-Rs with distinct kinetics, which differed among B cell subpopulations: switched memory cells rapidly upregulated TRAIL-R1 and -2 upon activation while naïve B cells only reached similar expression levels at later time points in culture. Increased expression of TRAIL-R1 and -2 coincided with a caspase-3-dependent sensitivity to TRAIL-induced apoptosis in activated B cells but not in freshly isolated resting B cells. Finally, both TRAIL-R1 and TRAIL-R2 could signal actively and both contributed to TRAIL-induced apoptosis. In conclusion, this study provides a systematic analysis of the expression of TRAIL-Rs in human primary B cells and of their capacity to signal and induce apoptosis. This dataset forms a basis to further study and understand the dysregulation of TRAIL-Rs and TRAIL expression observed in autoimmune diseases. Additionally, it will be important to foresee potential bystander immunomodulation when TRAIL-R agonists are used in cancer treatment.

## Introduction

The homeostasis of humoral immunity requires a tight control of generation, survival, activation and maturation of B lymphocytes. Therefore, pro-survival and pro-apoptotic signals play a very important role in shaping the B cell repertoire and the long-term memory compartment. Activation-induced cell death (AICD) is a key mechanism in the control of cell expansion and selection in the germinal center (GC), where B cells are activated and change their affinity for the antigen via somatic hypermutation (1). B cells with reduced specificity or with self-reactive B cell receptors should be eliminated (2, 3). Several members of the tumor necrosis factor (TNF) receptor superfamily have been implicated in the control of B cell homeostasis. Among these, Fas (CD95, Apo-1) is involved in the control of B cell activation and GC selection (4). Mutations in *Fas* lead to lymphoproliferation of B and T cells, and to autoimmunity (5, 6).

TNF-related apoptosis-inducing ligand receptor (TRAIL-R) 1 (aka DR4 or TNFRSF10A) and TRAIL-R2 (aka DR5 or TNFRSF10B) (7, 8) bind TRAIL and recruit downstream adaptor proteins via a conserved motif in the intracellular domain named death domain (DD), resulting in apoptosis. The system is regulated by 2 membrane bound decoy receptors: TRAIL-R3 (aka DCR1 or TNFRSF10C) and TRAIL-R4 (aka DCR2 or TNFRSF10D), that are devoid of a cytoplasmic tail or carry a truncated intracellular DD, respectively, and block TRAIL-mediated apoptosis (9–11). Also, the soluble TRAIL receptor osteoprotegerin (OPG or TNFRSF11B) can inhibit TRAIL-induced apoptosis (12) by modulating ligand availability. Furthermore, TRAIL-Rs may form heterodimers with each other or with other members of the TNF receptor superfamily, resulting in modulation of signaling responses (13–15).

Most of our knowledge on TRAIL-Rs expression and function derives from human cancer cell lines and mouse models. Mice express only one apoptosis inducing TRAIL-R (mTRAIL-R2) which is homologous to human TRAIL-R1 and -R2 (16) and two decoy receptors mDcTRAIL-R1 and mDcTRAIL-R2 along with OPG (17). Mouse mDcTRAIL-R1 and -R2 differ significantly in their amino acid sequence from their human counterparts and are devoid of any apoptotic or non-apoptotic signaling ability (17). Both, TRAIL and TRAIL-R deficient mice present a normally developed immune system. However, TRAIL-R deficient mice are characterized by dysregulated cytokine responses of innate immune cells (18). Furthermore, TRAIL and TRAIL-R deficient animals are more prone to tumor development (19, 20) and TRAIL deficient mice are more susceptible to induced autoimmunity (21). In Fas ligand (FasL) deficient mice, knockout of TRAIL exacerbates the FasL knockout phenotype, leading to extreme lymphoproliferation and fatal autoimmune thrombocytopenia (22), indicating that the TRAIL-R system partially functions as gatekeeper in absence of Fas signaling. As the number of receptors and the structure of decoy receptors are different, not all aspects of TRAIL-R biology can be transferred from mouse models to the more complex human system. In humans, TRAIL expression was described on various different innate and adaptive immune cell types including monocytes, macrophages, natural killer (NK) cells, T cells and B cells (23–26). TRAIL-R expression has been described in central and peripheral T cells and naïve and memory B cells upon activation (27, 28). While several non-transformed human cell types express TRAIL-Rs, many are refractory to the pro-apoptotic function of the ligand. Nevertheless, it has been shown that non-transformed cells can be sensitized to TRAIL-induced apoptosis by activating cues or viral infections (29–31). However, the results were depending on activation protocols and specific cellular subsets, leading to inconsistent conclusions (27, 28, 32, 33). A systematic description of TRAIL-Rs in human B cell subpopulations is missing, as well as a comprehensive analysis of the sensitivity of primary human B cells to TRAIL-induced apoptosis *ex vivo* and upon activation. Furthermore, the contribution of TRAIL-R1 and TRAIL-R2 to TRAIL-induced apoptosis in human B cells is largely unknown. Here, we provide a detailed expression profile of all TRAIL-Rs in primary human B cells derived from peripheral blood and secondary lymphoid organs. We show that GC B cells express significant levels of TRAIL-Rs and that different activation signals induced expression of all receptors accompanied by increased susceptibility to TRAIL-induced apoptosis. Furthermore, we show that in primary human B cells both TRAIL-R1 and TRAIL-R2 contribute to induction of apoptosis in a caspase-3-dependent manner.

## Materials and Methods

### Human Material

Buffy coats were purchased from the blood bank of the University Medical Center Freiburg (approval of the University Freiburg Ethics Committee: 147/15). Tonsillar cells from secondary lymphoid organs were obtained from biopsies of subjects without known immunodeficiency undergoing tonsillectomy (approval of the University Freiburg Ethics Committee: 121/11) and providing informed consent.

### Cell Isolation

PBMCs were isolated by density gradient centrifugation from buffy coats. B cells were isolated with the EasySep™ Human B cell Isolation Kit (Stemcell Technologies) following manufacturers' instructions. Mononuclear cells were isolated from tonsillar specimens by mechanical disruption. Minced tissue was pressed through a 380 μm meshed sieve and subsequently the cell suspension was filtered.

### Cell Culture

Magnetically isolated B cells at the concentration of 2.5–5 × 10^5^/mL were stimulated with CD40L and IL-21 as described (34), or with CpG ODN 2006 (Apara Biosciences, Germany) in Iscove's Modified Dulbecco's Medium (IMDM; ThermoFisher Scientific) supplemented with 10% FCS, insulin, apo-transferrin, non-essential amino acids, glutamine and glutathione as described earlier (35). TRAIL (SuperKillerTRAIL, Enzo Life Sciences), Fc-hFasL made as described (36), TRAIL-R1 blocking antibody (MA1-19025, clone DR-4-02, ThermoFisher Scientific), poly-caspase inhibitor Q-VD (R&D Systems), and Staurosporine (Sigma-Aldrich) were added at indicated concentrations.

The human Burkitt lymphoma cell line BJAB (purchased from Leibniz-Institut DSMZ—Deutsche Sammlung von Mikroorganismen und Zellkulturen GmbH) was cultured in IMDM supplemented with 10% FCS.

### Flow Cytometry

**Figure 1:**
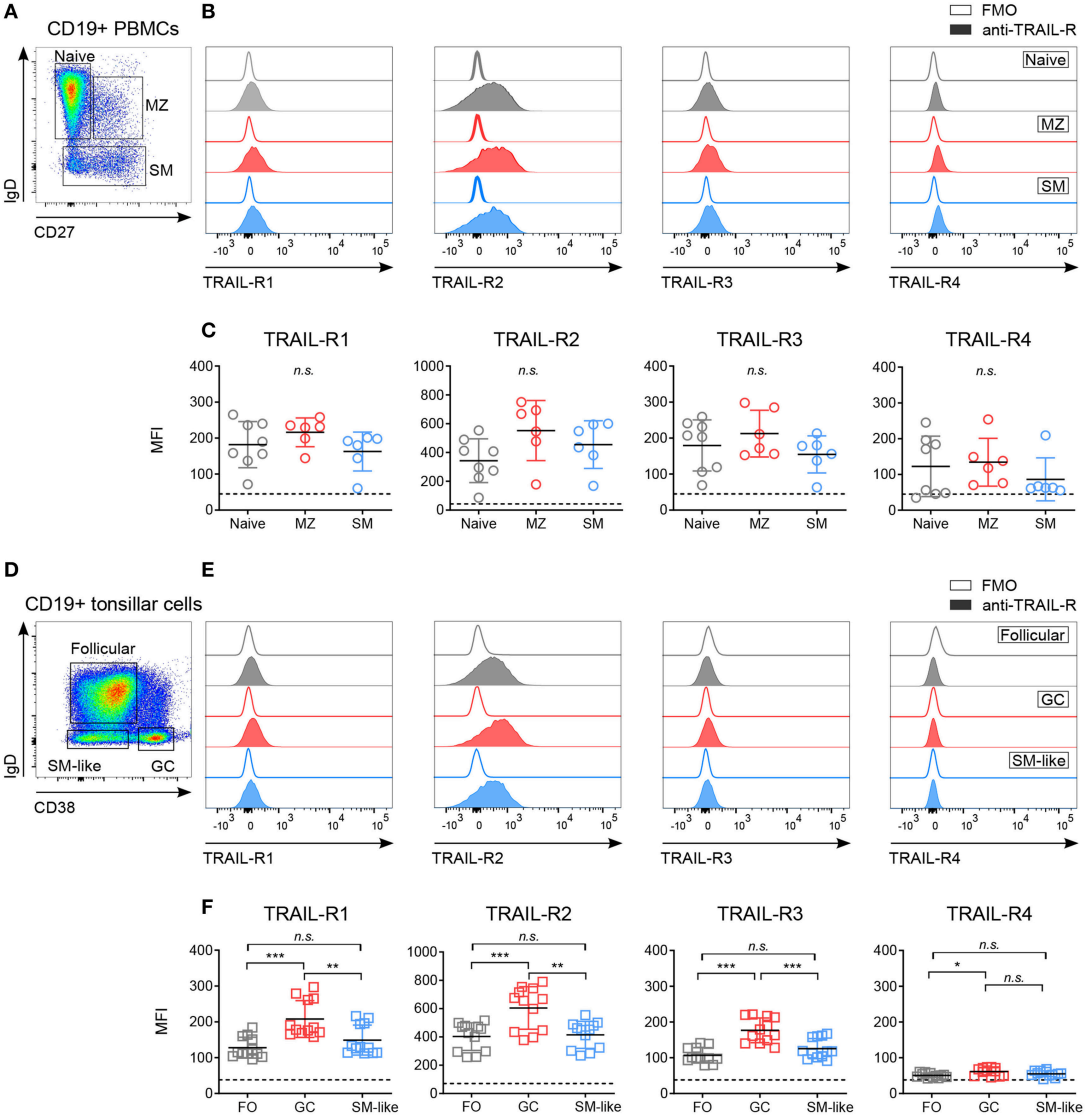
Differential expression of TRAIL-Rs in human B cell subpopulations. Expression of TRAIL-R1, TRAIL-R2, TRAIL-R3 and TRAIL-R4 was characterized by flow cytometry in *ex vivo* CD19^+^ human B cells isolated from PBMCs **(A–C)** and tonsillar CD19^+^ cells **(D–F)**. **(A)** Gating strategy to identify naïve (IgD^+^ CD27^−^), marginal zone (MZ; IgD^+^ CD27^+^) and switched memory (SM; IgD^−^ CD27^−^) cells from CD19^+^ live B cells. **(B)** Histograms show expression levels of indicated TRAIL-Rs in naïve, MZ and SM B cells compared to respective fluorescent minus one (FMO) controls. **(C)** Mean fluorescence intensity (MFI) of TRAIL-Rs among indicated B cell subsets. Each dot represents a biological replicate. **(D)** Gating strategy to identify follicular (FO; IgD^+^ CD38^−^), germinal center (GC; IgD^−^ CD38^+^) and switched memory-like (SM-like; IgD^−^ CD38^−^) cells among CD19^+^ live B cells. **(E)** TRAIL-R expression in FO, GC and SM-like B cells compared to respective FMO controls. **(F)** Quantifications of TRAIL-R expression among tonsillar B cell subpopulations depicted as MFI. Dots represent duplicates of six biological replicates. **(C,F)** Dashed lines indicate averaged FMO controls. Mean values ± SD are shown. *n.s*., not significant; ^*^adj.p < 0.05; ^**^adj.p < 0.01; ^***^adj.p < 0.001. Statistical analysis was performed using one-way ANOVA, followed by Tukey's multiple comparison test where appropriate.

Phenotype of human PBMCs and B cells was determined by flow cytometry with the following antibodies: CD16 PE (clone 3G8), mouse IgG1 PE isotype control (clone 678.1Mc7, all Beckman Coulter), IgD PE (polyclonal, Southern Biotech), CD95 PE (clone DX2), CD64 FITC (clone 10.1) CD19 PE-Cy7 (clone SJ25C1, all BD), CD19 APC-H7, CD19 BV421, CD19 BV510 (all clone HIB19), CD27 PE-Cy7 (clone M-T271), CD27 PerCP-Cy5.5 (clone O323), CD38 PE-Cy7 (clone HB-7), IgD PE-Cy7 (clone IA6-2), CD32 PE-Cy7 (clone FUN-2), mouse IgG1 APC isotype control (clone MOPC-21), TRAIL-R1 PE (307206, clone DJR1), TRAIL-R2 APC [307408, clone DJR2-4 (7–8)], TRAIL-R3 PE (307006, clone DJR3, all Biolegend) and TRAIL-R4 PE (A15779, clone TRAIL-R4-01, ThermoFisher Scientific). Dead cell exclusion was performed by 4′,6-diamidino-2-phenylindole (DAPI) staining or by Zombie NIR/ Aqua Fixable Viability Kit (both Biolegend). In some experiments cells were treated with Fc Receptor Blocking reagent (NB309; Innovex) according to manufacturers' instructions prior to surface staining to control for non-specific binding of antibodies to Fc receptors expressed on leukocytes. In blood derived samples we identified the following B cell subpopulations among the CD19^+^ alive single cell lymphocytes: Naïve B cells were defined as CD27^−^IgD^+^, marginal zone (MZ) B cells were defined as CD27^+^IgD^+^ and switched memory (SM) B cells were defined as CD27^+^IgD^−^ (Figure S1A, Figure 1A). Tonsillar CD19^+^live B cells can be categorized into follicular (FO) B cells, identified as CD38^−^IgD^+^ cells, SM-like B cells, defined as CD38^−^IgD^−^ and germinal center (GC) B cells, defined as CD38^+^IgD^−^ (Figure S1D, Figure 1D). Among GC B cells dark zone (DZ) B cells can be identified as CD83^−^CXCR4^+^ cells and light zone (LZ) B cells as CD83^−/+^CXCR4^−^ (37) (Figure S1E). Mean fluorescence intensity (MFI) of indicated markers was determined among total CD19/live B cells and subpopulations. Intracellular staining was performed using the IntraPrep Kit (Beckman Coulter) according to manufacturers' instructions. Activity of caspase-3 was detected by staining permeabilized B cells with an unconjugated rabbit anti-cleaved caspase-3 (Cell Signaling Technologies) antibody followed by the staining with a donkey anti-rabbit IgG PE antibody (Jackson ImmunoResearch). Stained cells were acquired using a FACS Canto II (BD Bioscience) and were analyzed with FlowJo (TreeStar Inc.).

### Generation of BJAB TRAIL-R2 Knockout Cell Lines

BJAB TRAIL-R2 knockout (KO) cell lines were generated by lentiviral delivery of a CRISPR-Cas9 vector system (38). The vector system expresses the gRNA, Cas9 protein and puromycin resistance gene. The gRNA was designed using the tool at http://crispor.tefor.net/ (39). The gRNA sequence used was 5′CGCGGCGACAACGAGCACAA3′ and respective oligos including adapters were cloned into the lentiCRISPR v2 vector (Addgene plasmid #52961) according to Zhang lab protocols (38). Successful cloning was confirmed by sequencing lentiCRISPR v2-TRAIL-R2 constructs. To produce lentiviral particles, packaging cell line 293T (purchased from Leibniz-Institut DSMZ—Deutsche Sammlung von Mikroorganismen und Zellkulturen GmbH) was co-transfected with PAX2 (Addgene plasmid #12260), pCMV-VSV-G (Addgene plasmid #8454) and lentiCRISPR v2-TRAIL-R2 plasmids using the jetPEI reagents (Polyplus-transfection) according to manufacturers' recommendations. Virus containing supernatant was harvested after 48 h and filtered through a 0.45 μm filter. For lentiviral transduction, BJAB cells were cultured with lentivirus containing medium supplemented with 8 μg/ml polybrene (Merck) for 24 h. Medium containing puromycin (Sigma-Aldrich) was added 72 h post-transduction. BJAB TRAIL-R2 KO cell lines were confirmed by flow cytometric analysis.

### Apoptosis Assay

To determine apoptosis in BJAB cells, 2.5 × 10^5^ cells/mL were seeded and after 24 h apoptosis-inducing agents TRAIL, Fc-hFasL, Staurosporine, and poly-caspase inhibitor Q-VD were added at indicated concentrations. In some experiments TRAIL-R1 mediated signaling was blocked by pre-incubating cells with a monoclonal TRAIL-R1 blocking antibody for 30 min at indicated concentrations. After overnight incubation, viability of cells was determined by DAPI staining.

To determine caspase-3 activation in response to TRAIL in activated cells, B cells were seeded at 2.5–5 × 10^5^ cells/mL and stimulated with CD40L and IL-21, apoptotic agents were added at indicated concentrations every day. After 4 h, cells were fixed and percentage of cleaved caspase-3^+^ cells was determined by flow cytometry. To identify apoptotic B cells after 24 h of TRAIL stimulation, cells were processed similarly with the exception that cells were stained with AnnexinV FITC (eBioscience) and DAPI according to manufacturers' instructions. Percentages of apoptotic cells were calculated by using the following formula:

$$\%\text{ of apoptotic cells}=\frac{\%\;A^{-}D^{-}\;\left(control\right)-\;\%\;A^{-}D^{-}\;(COI)}{\%\;A^{-}D^{-}\;\left(control\right)}*100\;$$

where % A^−^ D^−^ (control) represents the percentage of AnnexinV^−^DAPI^−^ cells of control cells cultured in normal cell culture medium and % A^−^ D^−^ (COI) represents the percentage of AnnexinV^−^DAPI^−^ cells cultured in the condition of interest.

### Quantitative PCR

Total RNA was isolated using TRIzol reagent (ThermoFisher Scientific) according to manufacturers' instructions. For cDNA synthesis, 100 ng RNA were reverse-transcribed using random hexamer primers (ThermoFisher Scientific) and SuperScript III reverse transcriptase (ThermoFisher Scientific). Quantitative RT-PCR was performed using the TaqMan Gene Expression Master Mix (ThermoFisher Scientific) and previously published primer/probe sets TNFRSF10A (TRAIL-R1), TNFRSF10B (TRAIL-R2), TNFRSF10C (TRAIL-R3) (40), TNFRSF10D (TRAIL-R4; all ThermoFisher Scientific) (41). All reactions were performed in duplicates and relative expression was calculated using the 2^−Δ*Cq*^ method with all mRNA levels normalized to the reference gene RPLPO (Hs99999902_m1, ThermoFisher Scientific).

### Statistics

Statistical analysis was performed with GraphPad Prism (GraphPad Software Inc.). Data are expressed as mean ± SD, SEM, or 10–90 percentile range as indicated. Unpaired two-tailed Student's *t*-test, one-way ANOVA, and two-way ANOVA were employed to test for statistical significance as indicated. Differences are indicated and were considered significant for *p* < 0.05.

## Results

### TRAIL-Rs Expression Is Modulated Along B Cell Development

While expression of CD40 and Fas has been studied extensively (4, 42–45), the expression of TRAIL-Rs in human B cell subpopulations has not been described in detail. We analyzed TRAIL-R1, TRAIL-R2, TRAIL-R3, and TRAIL-R4 in blood derived and tonsillar B cell subsets (Figure 1, Figure S1). Circulating B cell subsets expressed low but variable levels of all TRAIL-Rs, without significant differences between naïve, marginal zone (MZ), and switched memory (SM) B cells (Figures 1A–C, Figure S1A). As *ex vivo* expression of the receptors was low, we performed control experiments to ensure specific TRAIL-R antibody staining and exclude Fc receptor mediated aspecific binding of antibodies. We analyzed TRAIL-R expression of B cells *ex vivo* after Fc receptor blocking and compared isotype to fluorescent minus one (FMO) controls (Figures S1A,B) and observed that staining of TRAIL-Rs was not influenced by nonspecific antibody binding. In line with that, resting CD19^+^ B cells did not express high affinity Fcγ receptor CD16 and low affinity Fcγ receptor CD64, albeit they were positive for low affinity Fcγ receptor CD32 compared to myeloid CD33^+^ cells (Figure S1C). *In vivo* activated GC derived B cells were analyzed in tonsillar samples and we observed increased expression of TRAIL-R1 and especially of TRAIL-R2 in GC B cells compared to follicular (FO) and SM-like B cells (Figures 1D–F, Figure S1D). Interestingly, FO and SM-like B cells showed similar TRAIL-R1 and TRAIL-R2 expression levels to their blood derived naïve and SM counterparts. TRAIL-R3 was increased in GC B cells compared to FO and SM-like B cells whereas TRAIL-R4 was very low in all tonsillar B cell subsets, with GC B cells showing the highest expression (Figure 1F). GC B cells can be further delineated phenotypically and functionally into dark zone (DZ) and light zone (LZ) B cells (37, 46) (Figure S1E). Expression of all TRAIL-Rs was similar between DZ and LZ B cells (Figure S1F). Differential expression of TRAIL-Rs in tonsillar B cells suggested a unique and distinct susceptibility of different B cell subpopulations to TRAIL. Increased expression of pro-apoptotic TRAIL-Rs in GC B cells may suggest a role of TRAIL mediated apoptosis during GC B cell selection.

### TRAIL-Rs Expression Is Induced Upon T Cell-Dependent and T Cell-Independent Activation of B Cells

**Figure 2:**
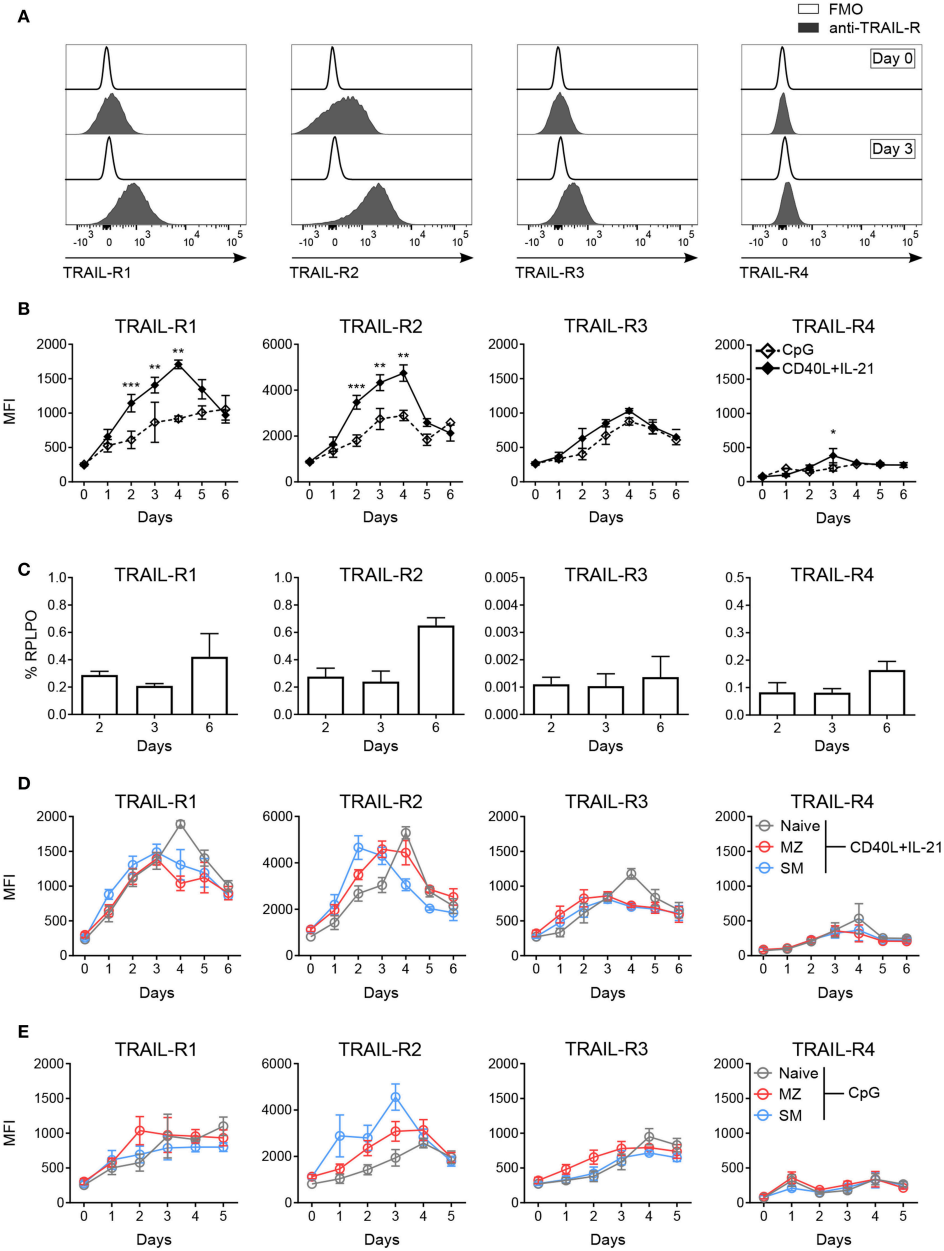
*In vitro* activation induces TRAIL-R expression in human B cells. Expression of TRAIL-Rs was analyzed by flow cytometry and RT-qPCR in *in vitro* activated human B cells isolated from PBMCs. **(A)** Representative histograms depict expression of TRAIL-Rs in CD19^+^ live B cells at day 0 and at day 3 after stimulation with CD40L+IL-21 compared to respective fluorescent minus one (FMO) controls. **(B)** Quantification of TRAIL-R expression in CD19^+^ live B cells upon stimulation with CpG or CD40L+IL-21 up to day 6 of culture depicted as MFI (*n* ≥ 3). **(C)** RT-qPCR analysis of all TRAIL-R transcripts in isolated CD19^+^ B cells upon stimulation with CD40L+IL-21 at day 2, 3 and 6 (*n* = 3). **(D)** TRAIL-R expression in naïve, MZ and SM B cells upon T cell-dependent (CD40L+IL-21) activation up to day 6 of culture shown as MFI (*n* ≥ 3). **(E)** TRAIL-R expression in naïve, MZ and SM B cells upon T cell-independent (CpG) stimulation up to day 5 of culture depicted as MFI (*n* ≥ 3). Mean values ± SEM **(B,D,E)** or ± SD **(C)** are shown. *n*, biological replicates. **(B)** ^*^p < 0.05; ^**^p < 0.01; ^***^p < 0.001. Statistical analysis was done with two-tailed unpaired Student's *t*-test.

Sensitivity to apoptosis mediated by receptor-ligand pairs of the TNF superfamily strongly depends on the expression of respective receptors (45, 47). We monitored expression levels of TRAIL-R1, TRAIL-R2, TRAIL-R3, and TRAIL-R4 in blood derived B cells upon *in vitro* activation. T follicular helper (T_FH_) cell-dependent stimulation with CD40L and IL-21 strongly induced expression of TRAIL-R1, TRAIL-R2 and TRAIL-R3, while TRAIL-R4 remained mostly unchanged (Figures 2A,B). TRAIL-Rs staining was not influenced by unspecific antibody binding to Fc receptors (Figure S2A) and Fcγ receptor expression (CD16, CD32, CD64) was not affected by activation (Figure S2B). The four TRAIL receptors showed different kinetics of expression: TRAIL-R1, TRAIL-R2, and TRAIL-R3 showed a maximal expression at day 4, but while TRAIL-R1 and -R2 sharply declined until day 6, TRAIL-R3 remained more stable (Figure 2B). Expression of TRAIL-R4 was only weakly, incrementally induced until day 6 (Figure 2B). We next performed qPCR for all TRAIL-R transcripts in isolated CD19^+^ B cells in response to CD40L and IL-21 and found that all TRAIL-R transcripts were expressed (Figure 2C). Interestingly, transcription levels for all receptors were stable until day 3 and only increased at day 6, suggesting that the initial increase in membrane protein expression was due to already present intracellular reservoirs of translated protein [reviewed in (48)]. T cell-independent activation, mediated by toll-like receptor 9 (TLR9) agonist resulted in weaker, delayed, albeit more stable expression of all TRAIL-Rs (Figure 2B). We found differences in the induction of single TRAIL-Rs among B cell subpopulations in response to T_FH_-dependent stimulation as SM B cells reached the maximal expression of TRAIL-R2 after 2 days of stimulation, much quicker compared to MZ and naïve B cells (Figure 2D, Figure S2C). Naïve B cells reached the peak of expression of TRAIL-R2 at day 4, when TRAIL-R2 on memory cells already started to decline (Figure 2D, Figure S2C). Although TRAIL-Rs expression upon activation was delayed in naïve B cells, expression reached a level comparable to SM and MZ B cells (Figure 2D), explaining the discrepancy with published data showing a high expression of TRAIL-Rs in memory B cells vs. naïve B cells after 2 days of activation (27). The dynamic of TRAIL-Rs expression in B cell subpopulations in response to TLR9 stimulation revealed a rapid increase of TRAIL-R2 in memory B cells, already visible at day 1, and a slower and steady increase in TRAIL-R1 (Figure 2E). As expected, naïve B cells were poorly responsive to TLR9 stimulation at early time points of culture, resulting in an inefficient induction of TRAIL-Rs (Figure 2E).

### TRAIL Induces Apoptosis in Activated but Not in Resting Primary Human B Cells

**Figure 3:**
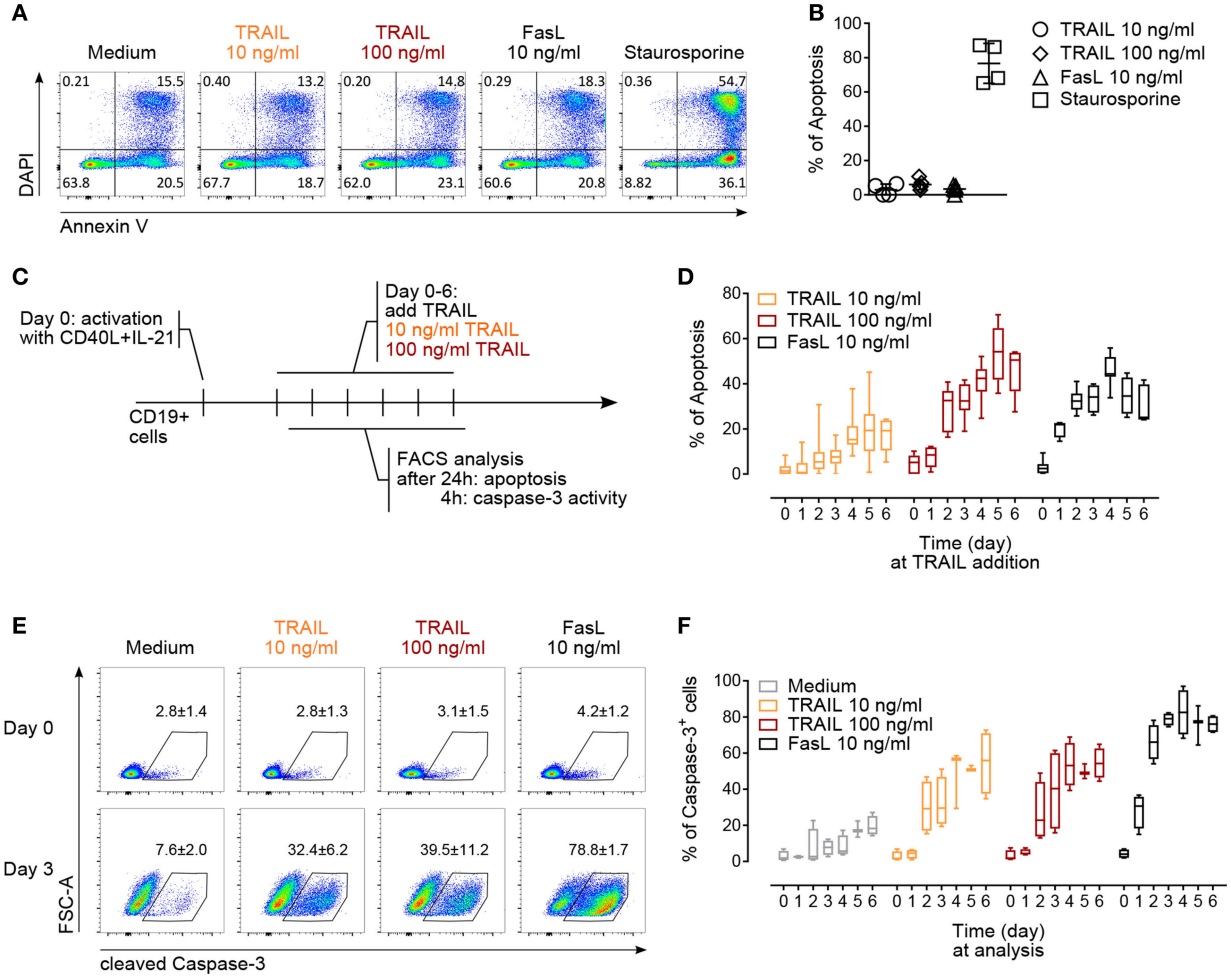
TRAIL induces caspase-3 activation and promotes apoptosis in activated human B cells. **(A)** Apoptosis of resting CD19^+^ B cells in response to TRAIL, FasL or staurosporine (1 μM) for 24 h was determined by Annexin V and DAPI staining and flow cytometric analysis. **(B)** Percentage of apoptotic resting CD19^+^ B cells after incubation for 24 h with TRAIL, FasL or staurosporine was quantified by flow cytometry. The percentage of apoptotic B cells is shown, see Material and Methods for formula. Each dot represents a biological replicate. **(C)** Experimental setup to analyze TRAIL-induced apoptosis and caspase-3 induction in T cell-dependent activated B cells by flow cytometry. **(D)** Apoptosis was quantified after 24 h of TRAIL or FasL stimulation of activated B cells until day 6 of culture, as described in **(C)**. The percentage of apoptotic B cells is shown (*n* ≥ 6). **(E)** Representative flow cytometric analysis of FSC-A and cleaved caspase-3 in resting and 3-days *in vitro* activated CD19^+^ B cells after 4 h of TRAIL or FasL treatment. Numbers reflect mean percentage of cleaved caspase-3^+^ cells ± SEM **(F)** percentages of caspase-3^+^ B cells after 4 h of TRAIL or FasL stimulation in activated B cells until day 6 of culture (*n* ≥ 3). **(B,E)** Mean values ± SD or SEM are shown, respectively. **(D,F)** Data are expressed as 10–90 percentile.

The regulation of AICD in T and B lymphocytes is crucial to maintain peripheral immune tolerance and is mainly attributed to Fas (4, 47, 49). Based on the observed increased expression of TRAIL-Rs upon *in vitro* activation, we hypothesized that TRAIL may contribute to AICD in human B cells. Resting B cells were resistant to TRAIL- or FasL-induced apoptosis (Figures 3A,B). On the contrary, *in vitro* CD40L and IL-21 activated B cells became gradually susceptible to both TRAIL- and FasL-mediated apoptosis until day 6 of culture (Figures 3C,D). The highest percentage of apoptosis was observed between day 4 to day 6 (Figure 3D), corresponding to the highest expression of TRAIL-R1 and TRAIL-R2 at day 4 and the still high expression of TRAIL-R1 at day 5 and day 6 (see Figure 2B), suggesting that TRAIL indeed may contribute to AICD in human B cells. Death receptors of the TNF-R superfamily have been shown to employ and recruit multiple adaptor and signaling molecules to induce apoptosis. To identify the role of caspases in TRAIL-mediated apoptosis in primary human B cells we analyzed up to day 6 of culture the progressive ability of TRAIL to induce the activation of executioner caspase-3 after 4 h of TRAIL-R ligation (Figure 3C). B cells activated with CD40L and IL-21 were more prone to TRAIL-mediated activation of caspase-3 than resting B cells (Figure 3E). Furthermore, caspase-3 activation increased progressively until day 4 to day 6 of culture, in line with their sensitivity to TRAIL-induced apoptosis (Figure 3F). Together, these data provide evidence that T cell-dependent activation induces expression of TRAIL-Rs in primary human B cells and renders them susceptible to TRAIL-induced caspase-3 activation and apoptosis. In this setting B cells were first stimulated with CD40L and IL-21 and then, delayed in time (days 1–6), TRAIL was added to the culture. This justifies the discrepancy with the report of CD40L-mediated resistance to TRAIL-induced apoptosis in human tonsillar and malignant B cells where CD40L and TRAIL were added simultaneously to the B cells (50).

### Both TRAIL-R1 and TRAIL-R2 Contribute to Apoptosis in Primary Human B Cells and Tumor B Cell Lines

**Figure 4:**
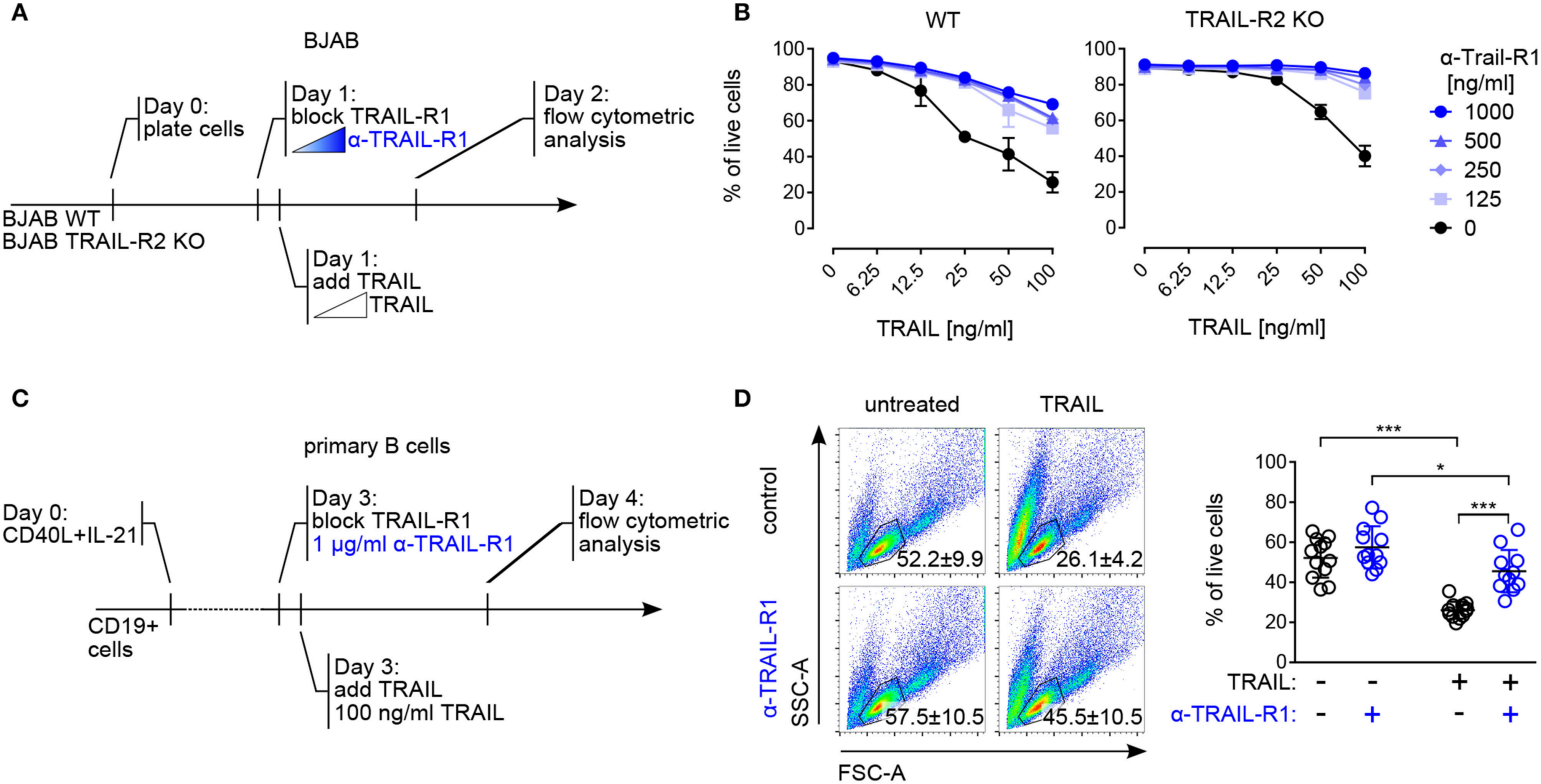
Both TRAIL-R1 and TRAIL-R2 mediate apoptosis in primary human B cells and tumor B cell lines. **(A)** Experimental approach, describing the addition of reagents to the culture to study TRAIL-induced apoptosis in BJAB cells by flow cytometry. **(B)** Percentages of live BJAB WT (left) and TRAIL-R2 KO (right) cells after 24h of scalar concentrations of TRAIL ± scalar concentrations of TRAIL-R1 blocking antibody (*n* = 3). **(C)** Experimental setup to analyze contribution of TRAIL-R1 and TRAIL-R2 to TRAIL-induced apoptosis in activated B cells by flow cytometry. **(D)** Representative identification of live primary B cells by FSC-A and SSC-A (left) and quantification (right) of live B cells after 24 h of TRAIL stimulation ± TRAIL-R1 blocking antibody (1μg/ml) at day 4 of culture by flow cytometry. Dots represent triplicates of four biological replicates. (B, D) Mean values ± SD are shown. **(B)** *n*, independent experiments. **(D)** ^*^adj.*p* < 0.05; ^***^adj.*p* < 0.001. Statistical analysis was performed using two-way ANOVA, followed by Tukey's multiple comparison test where appropriate.

Cell death induced by both TRAIL-R1 and TRAIL-R2 has been implicated in the control of human B cell lymphoma, with a specific contribution of each receptor, dependent on their relative surface expression (51) Indeed, in line with published data (52, 53) the human Burkitt lymphoma-derived B cell line BJAB expressed both TRAIL-R1 and TRAIL-R2 (Figure S3A) and was sensitive to TRAIL-mediated apoptosis (Figures 4A,B). Pre–incubation with scalar concentrations of a TRAIL-R1 blocking monoclonal antibody inhibited TRAIL-induced apoptosis in BJAB cells progressively but did not fully abrogate cell death (Figures 4A,B). Importantly, the TRAIL-R1 blocking antibody completely inhibited TRAIL-induced killing in BJAB knockout for TRAIL-R2 (BJAB TRAIL-R2 KO) (Figure 4B, Figure S3A). Based on these data we confirmed that both TRAIL-R1 and TRAIL-R2 contributed to the apoptosis control of tumor B cell lines. Additionally, we validated the use of TRAIL-R1 blocking antibody as an appropriate reagent to study the relative contribution of TRAIL-R1 and TRAIL-R2 to apoptosis. Next, we assessed the contribution of TRAIL-R1 and TRAIL-R2 to TRAIL-mediated apoptosis in primary human B cells. To this end, we exposed B cells activated for 4 days with T_FH_-like cues to TRAIL-R1 blocking antibody in combination with TRAIL and analyzed cell death after 24 h (Figure 4C). TRAIL treatment significantly reduced the percentage of living cells in a caspase-dependent manner (Figure S3B). Pre-incubation with TRAIL-R1 blocking antibody increased B cell survival upon TRAIL incubation significantly, from 26 to 45% (Figure 4D). However, TRAIL-R1 inhibition did not result in a complete abrogation of cell death, as untreated cells showed 57% of survival (Figure 4D). When TRAIL-R1 is blocked by the specific TRAIL-R1 antibody the only receptor competent to induce apoptosis is TRAIL-R2. The significant differences observed in TRAIL-R1 blocked cells following TRAIL incubation (57 to 45%) indicate a contribution of TRAIL-R2 to TRAIL-induced apoptosis in primary human B cells. Thus, primary B cells are susceptible to apoptosis by ligand-induced crosslinking of both TRAIL-R1 and TRAIL-R2 (Figure 4D).

## Discussion

Activation-induced apoptosis is one of the key mechanisms to control proliferation and selection of B lymphocytes (54, 55). While the role of Fas has been addressed by several studies in this context, the impact of the TRAIL-R system on human B lymphocytes has been studied insufficiently. Importantly, the knowledge we have from mouse models can be poorly transferred to the human system as the number of TRAIL receptors and the structure of the decoy receptors are different in the two species. Hence, we undertook the task to systematically describe TRAIL-Rs expression in all developmental stages of human B cells isolated from blood and secondary lymphoid organs from naïve to SM B cells. We studied the sensitivity to apoptosis in response to TRAIL, the activation of effector caspases and the respective contribution of TRAIL-R1 and TRAIL-R2 to the apoptosis induction in primary human B cells.

We found that the expression of TRAIL-Rs is modulated along development, with GC B cells expressing highest levels of all TRAIL receptors compared to all other B cell subpopulations. Our data are in contrast with a previously published report showing that TRAIL-R2 expression is restricted to monocytes and TRAIL-R1 to B lymphocytes (56). This discrepancy may be due to different assay sensitivities, employing different antibodies to stain TRAIL-Rs, as well as to the fact that the analysis of single subpopulations may increase the detection limit of the assay. We used fluorescence minus one staining to assess background intensity and to identify stained cells, also at low levels of signal intensity, and we excluded aspecific staining due to Fc receptor binding of staining antibodies. Indeed, peripheral B cells show lower levels of TRAIL-R staining compared to GC B cells, but still distinguishable from background. Moreover, we showed that TRAIL-Rs are induced upon activation via CD40 and IL-21 receptor, with a kinetic specific to each subpopulation, with memory cells showing the fastest and highest expression of TRAIL-R1 and TRAIL-R2. The ability of CD40 or B cell receptor stimulation to induce TRAIL-R1 and TRAIL-R2 was shown previously (27), but the expression of the receptors was checked only 2 days after activation. We showed that naïve B cells reached the same level of TRAIL-Rs expression as memory B cells, only after a longer activation period. For the first time, we showed that innate-like stimuli via TLR9 can induce TRAIL-Rs expression in B cells with a distinct kinetic characterized by a weaker and more sustained expression compared to T_FH_-dependent activation, and a fast induction of TRAIL-R2 in memory B cells. The determination of the expression levels of receptors and their decoy is important for several reasons.

First, in other cellular systems it has been shown that combinatorial receptor expression corresponded to their susceptibility to apoptosis in response to TRAIL. Indeed, it was shown that human resting monocytes and *in vitro*-differentiated macrophages expressed substantial levels of TRAIL-R1 and TRAIL-R2, and neutrophils TRAIL-R3. Accordingly, exclusively monocytes and macrophages activated caspase-8 and underwent apoptosis upon recombinant TRAIL treatment (57). In contrast with these data, we found that B cells expressing TRAIL-R1 and TRAIL-R2 were resistant to TRAIL-induced apoptosis *ex vivo*, but became sensitive upon activation, when their surface receptor levels increased up to 10-fold.

Second, it has been reported that different members of the TNF receptor superfamily can heteromerize, thereby modulating their ligand induced signaling responses. In particular, in B cells CD40-TRAIL-R2 association results in reduced CD40-mediated NF-κB signaling (15). Hence, as CD40 expression is constant along B cell development the modulation of TRAIL-R2 expression that we observed might directly result in an intrinsic difference in response to CD40 activation in B cells.

Third, in line with the resistance of B cells to TRAIL-induced apoptosis *ex vivo*, it has been reported that TRAIL acts as an apoptosis inducer for cancer cells sparing non-tumor cell targets. The sensitivity of tumor cells to TRAIL is associated with the constitutive embedding of TRAIL-R1 in lipid micro domains, whereas its exclusion was associated with TRAIL resistance. These data, derived from the study of chronic lymphocytic leukemia, show that not only the expression but also the location of the receptor within the membrane controls the susceptibility to apoptosis induced by TRAIL. Notably, the sole recruitment of TRAIL-R1 and not TRAIL-R2 was linked to apoptosis of the cells in this study (58). Indeed, recent studies show that the plasma membrane of B lymphocytes is highly compartmentalized (59–61), and the compartmentalization of the receptors changes upon activation, contributing to modulation of signaling. Unfortunately, the location of TRAIL-Rs within the plasma membrane in resting and activated B cells is not known. Nevertheless, our observation that activation of B cells renders them susceptible to TRAIL-induced apoptosis, tempts us to hypothesize that relocation of the TRAIL receptors may contribute to this phenomenon. Alternatively, different oligomeric requirements may be needed to trigger apoptotic signals in activated or resting cells as it was shown in different tumor cell lines (62, 63).

We show that activated B cells in a T cell-dependent manner (CD40L+IL21) are progressively sensitive to TRAIL-induced apoptosis, which correlates with the increased expression of TRAIL-R1 and TRAIL-R2 following activation. These observations are opposing to the reported CD40L-mediated resistance to TRAIL-induced apoptosis in human follicular lymphoma B cells and normal tonsillar B cells (50). In this study TRAIL-induced apoptosis was inhibited by concomitant stimulation with CD40L and attributed to CD40-induced activation of the NF-κB1 pathway. In fact, protection from Fas or TRAIL-R mediated apoptosis by simultaneous CD40 ligation has been reported (64–66). Here, we studied TRAIL-induced apoptosis in blood-derived B cells which received T cell-dependent activation cues (i.e., CD40L and IL-21) at earlier time points, which allowed us to test intrinsic responses of B cells expressing significant levels of pro-apoptotic TRAIL-Rs to TRAIL. Our findings are important because during GC B selection *in vivo*, pro-survival signals like CD40L and IL-21 are limiting and only transiently provided by T_FH_ cells (46, 67). Interestingly, we found that GC B cells have the highest expression of TRAIL-Rs among tonsillar B cells. It has been shown that both activated T and B cells express TRAIL (68), as well as NK cells, dendritic cells and macrophages (69). Taken together, these data suggest that TRAIL-induced apoptosis may contribute to the control of B cell selection and GC homeostasis. This hypothesis is supported by data acquired from mice carrying double knockout for TRAIL and FasL resulting in a more severe lymphoproliferation and accelerated thrombocytopenia when compared to FasL knockout only animals (22). There are no available data on the expression and function of TRAIL-Rs in autoimmune lymphoproliferative syndrome caused by *Fas* mutations. Noteworthy, mutations in *caspase-10* (70) or *caspase-8* (71), both downstream of Fas and TRAIL-R signaling, result in immune dysregulation with features of autoimmune lymphoproliferative syndrome. Moreover, caspase-8 deficiency (71) is associated with defects in B and T cell activation and immunodeficiency whereas caspase-10 deficiency is associated with susceptibility to infections (72) and arthritis (73), pointing to a dual role of caspases in inducing both apoptotic and non-apoptotic signaling. Indeed, the real contribution of TRAIL and TRAIL-R expression in autoimmune disease is still unclear, as the available data are controversial. For example, decrease of TRAIL-R2 on CD8 T cells was reported in severe aplastic anemia (74), conversely, in rheumatoid arthritis TRAIL and its receptors (both death and decoy) were increased in both CD4 and CD8 T cells compared to control individuals, and the variation of TRAIL-R1 on CD8 T cells correlated with the disease activity (75). Hence, the dysregulation of TRAIL-Rs observed in autoimmunity can point both to impaired apoptosis, as well as to compromised non-apoptotic signaling and lymphocytes activation.

We analyzed the contribution of TRAIL-R1 and TRAIL-R2 to TRAIL-induced apoptosis in activated primary human B cells, and found that both receptors are involved, with TRAIL-R1 being the major contributor. These data are important as TRAIL specifically induces apoptosis in multiple tumor cell lines, which motivated the clinical development of several agonists of TRAIL-Rs, especially of TRAIL-R2. Even though, to date, recombinant forms of TRAIL, and agonistic antibodies against TRAIL-R1 and TRAIL-R2 have failed to provide clinical benefit in cancer patients, novel cross-linking agonistic TRAIL-R antibodies are currently under evaluation (76, 77) and different approaches have been undertaken to re-sensitize transformed cells to TRAIL (31, 62, 78). It became clear that a specific agonist of TRAIL-R1 or TRAIL-R2 will not have an effect only on tumor cells, but also on normal cells, possibly inducing apoptosis on normal GC activated B cells for example. We could expect that this would have implications on B cell selection, antibody responses to antigen and formation of memory cells and plasmablasts, to the extent of inducing an immunodeficiency.

In conclusion, our study provides a systematic analysis of the expression, signaling capacity and induction of apoptosis of TRAIL-Rs in primary human B cells. The results represent the starting point for the understanding of the dysregulation of TRAIL-Rs and TRAIL expression observed in autoimmune diseases. Additionally, these data are important to foresee potential bystander immunomodulation occurring on normal cells when TRAIL-R agonists are used in cancer treatment.

## Ethics Statement

Buffy coats were purchased from the blood bank of the University Medical Center Freiburg (approval of the University Freiburg Ethics Committee: 147/15). Tonsillar cells from secondary lymphoid organs were obtained from biopsies of subjects without known immunodeficiency undergoing tonsillectomy (approval of the University Freiburg Ethics Committee: 121/11) and providing informed consent.

## Author Contributions

JS, RL, BH, and IJ performed experiments. SU, KW, and MS prepared lymph nodes material. PS provided key reagents. JS and MR analyzed data. JS, PS, NV, JT, CS, and MR discussed the data and wrote the manuscript.

### Conflict of Interest Statement

The authors declare that the research was conducted in the absence of any commercial or financial relationships that could be construed as a potential conflict of interest.
