# Supplementary material for: TRAIL-R1 and TRAIL-R2 Mediate TRAIL-Dependent Apoptosis in Activated Primary Human B Lymphocytes
Source: Front Immunol. 2019 Apr 30;10:951. doi: 10.3389/fimmu.2019.00951 (PMC6503035; doi:10.3389/fimmu.2019.00951)
Supplement: Supplementary file 1 [file Data_Sheet_1.pdf]

‘TRAIL-R1 and TRAIL-R2 mediate TRAIL-dependent apoptosis in activated primary human B lymphocytes’ Staniek et al

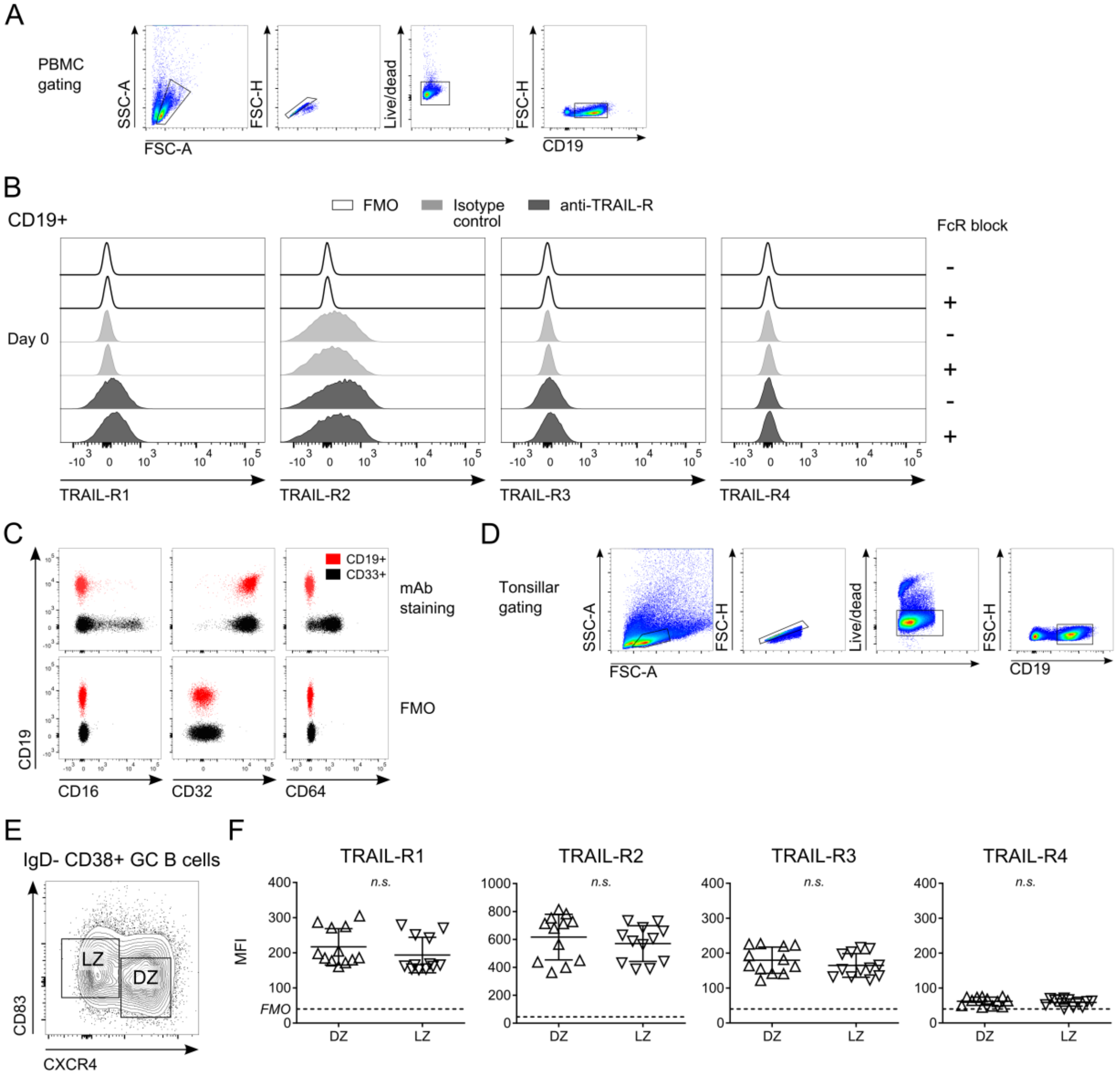

Supplementary Figure 1

Figure S1

Identification of CD19$^+$ live cells from PBMCs and tonsillar specimens and expression of TRAIL-Rs and Fcγ receptors *ex vivo*. (A) Gating strategy to identify B cells from PBMCs. (B) Representative histograms show expression of indicated TRAIL-Rs in CD19$^+$ live cells from PBMCs with and without prior saturation of Fcγ receptors compared to fluorescent minus one (FMO) and isotype controls. (C) Expression of Fcγ receptors CD16, CD32, CD64 in CD19$^+$ and CD33$^+$ cells *ex vivo*. (D) Gating strategy used to identify B cells from tonsils. (C) DZ (CD83- CXCR4+) and LZ (CD83+/- CXCR4-) identified from IgD- CD38+ GC B cells. (D) Quantification of TRAIL-R expression in DZ and LZ B cells depicted as MFI. Dots represent duplicates of six biological replicates. Dashed lines indicate averaged FMO controls. Mean values ± SD are shown. n.s., not significant. Statistical analysis was done with two-tailed unpaired Student’s t-test.

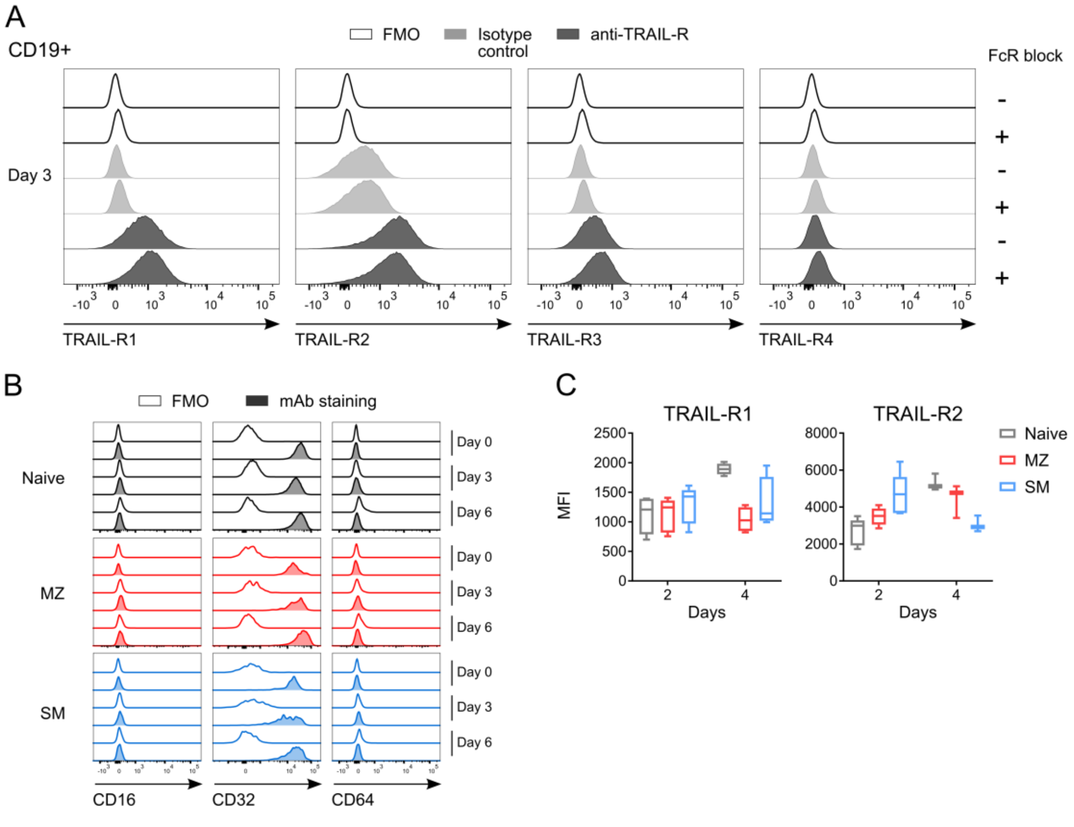

Supplementary Figure 2

Figure S2

Flow cytometric analysis of TRAIL-Rs and Fcγ receptors in CD19$^+$ live cells activated with CD40L+IL-21. (A) Representative histograms show expression of indicated TRAIL-Rs compared to fluorescent minus one (FMO) and isotype controls in activated CD19$^+$ live cells at day 3. TRAIL-Rs were stained with and without previous blocking of Fcγ receptors. (B) Expression of Fcγ receptors CD16, CD32, CD64 was assessed in activated naïve, MZ and SM B cells at day 3. (C) Expression of TRAIL-R1 and TRAIL-R2 in CD40L+IL21 activated primary B cells at day 2 and day 4 of culture. TRAIL-R MFI values of naïve, MZ and SM B cells are expressed as 10-90 percentile (n≥4).

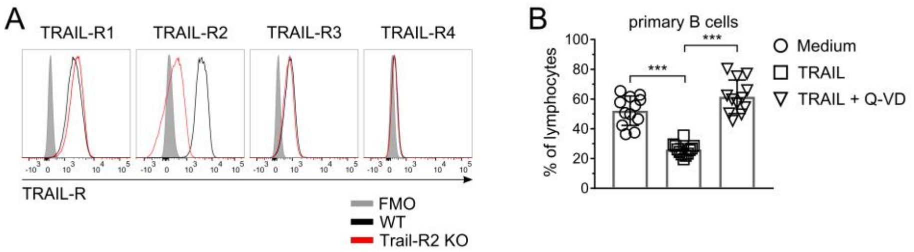

Supplementary Figure 3

Figure S3 refers to Figure 4

TRAIL-R phenotyping of BJAB WT and BJAB TRAIL-R2 KO cells and TRAIL-induced apoptosis in primary human B cells. (A) Expression of indicated TRAIL-Rs in BJAB WT and BJAB TRAIL-R2 KO cells. (B) Percentage of live B cells after 24h of TRAIL stimulation ± poly-caspase inhibitor Q-VD (20 µM) at day 4 of culture by flow cytometry. Dots represent triplicates of four biological replicates. Mean values ± SD are shown. ****adj.p*<0.001. Statistical analysis was performed using two-way ANOVA, followed by Tukey's multiple comparison test where appropriate.
